# Effect of Topical Administration of Somatostatin on Retinal Inflammation and Neurodegeneration in an Experimental Model of Diabetes

**DOI:** 10.3390/jcm9082579

**Published:** 2020-08-10

**Authors:** Cristina Hernández, Ana I Arroba, Patricia Bogdanov, Hugo Ramos, Olga Simó-Servat, Rafael Simó, Angela M Valverde

**Affiliations:** 1 Diabetes and Metabolism Research Unit. Vall d’Hebron Research Institute (VHIR) and Autonomous University of Barcelona (UAB), 08035 Barcelona, Spain; patricia.bogdanov@vhir.org (P.B.); hugo.ramos@vhir.org (H.R.); olga.simo@vhir.org (O.S.-S.); rafael.simo@vhir.org (R.S.); 2 Spanish Biomedical Research Centre in Diabetes and Associated Metabolic Disorders (CIBERdem), ISCIII, 28029 Madrid, Spain; ana.arroba@inibica.es (A.I.A.); avalverde@iib.uam.es (A.M.V.); 3 Research Unit, Instituto de Investigación e Innovación en Ciencias Biomédicas de la Provincia de Cádiz (INiBICA), University Hospital “Puerta del Mar”, 11570 Cádiz, Spain; 4 Alberto Sols Biomedical Research Institute (IIBm) (CSIC/UAM), 28029 Madrid, Spain

**Keywords:** somatostatin, retinal neurodegeneration, retinal inflammation, diabetic retinopathy, microglia, db/db mice

## Abstract

Somatostatin (SST) is a neuroprotective peptide but little is known regarding the potential role of its anti-inflammatory effects on retinal neuroprotection. In a previous study, we provided the first evidence that topical (eye drops) administration of SST prevents retinal neurodegeneration in streptozotocin (STZ)-induced diabetic rats. However, STZ by itself could cause neurotoxicity, thus acting as a confounding factor. The aims of the present study were: (1) to test the effect of topical administration of SST in the db/db mouse model, a spontaneous model of type 2 diabetes, thus avoiding the confounding effect of STZ on neurodegeneration; (2) to further explore the anti-inflammatory mechanisms of SST in glial cells. This task was performed by using mouse retinal explants and cell cultures. In summary, we confirm that SST topically administered was able to prevent retinal neurodysfunction and neurodegeneration in db/db mice. Furthermore, we found that SST prevented the activation of the classical M1 response of Bv.2 microglial cells upon Lipopolysaccharide (LPS) stimulation as a potent pro-inflammatory trigger. The anti-inflammatory effect of SST in Bv.2 cells was also observed in response to hypoxia. In conclusion, we provide evidence that the neuroprotective effect of SST in diabetic retinas can be largely attributed to anti-inflammatory mechanisms.

## 1. Introduction

Several reports indicate that somatostatin (SST) is locally produced by the retina in relatively abundant amounts and plays a key role in maintaining retinal normal homeostasis [1,2,3]. In diabetic retinopathy (DR), there is a downregulation of retinal expression of SST [4], which is associated with a dramatic decrease in intravitreal SST levels in both Proliferative Diabetic Retinopathy (PDR) [2,5] and Diabetic Macular Edema (DME) [6]. This decrease in SST levels locally produced by the retina has been involved in the pathogenesis of DR [2]. Therefore, SST replacement treatment by using eye drops can be envisaged as a reasonable approach for treating DR. In this regard, we provided the first evidence that topical administration of SST prevents retinal neurodegeneration in streptozotocin (STZ)-induced diabetic rats [7] and arrests the progression of neurodysfunction in subjects with type 2 diabetes [8].

Murine models based on STZ administration that mimic type 1 diabetes are currently used to investigate retinal abnormalities induced by diabetes. The glucose moiety in its chemical structure enables STZ to enter the beta cell via GLUT-2 glucose transporter, causing toxicity [9]. It must be noted that GLUT-2 is also present in the murine retina [10] and, particularly, it is expressed in Müller cells. For this reason, a debate has arisen regarding the appropriateness of this model for examining retinal neurodegeneration shortly after STZ administration. In fact, low doses of STZ injected into the lateral cerebral ventricles lead to structural and functional changes in the brain that resemble features of patients with Alzheimer disease [11,12]. In addition, direct histopathological evidence of specific neurotoxic damage caused by intracerebroventricular STZ administration in the fornix, anterior hippocampus and periventricular structures has been reported [13]. For all these reasons, the use of a spontaneous model of diabetes seems recommendable for investigating the underlying mechanisms of retinal neurodegeneration associated with diabetes and for testing new drugs. In this regard, we previously reported the functional and structural damage of the retina in db/db mouse [14]. The db/db mouse carries a mutation in the leptin receptor gene and is a well-established model of obesity-induced type 2 diabetes.

There are several studies on the mechanisms involved in the neuroprotective action of SST in the retina [15,16,17], but little is known regarding the potential role of its anti-inflammatory effects on retinal neuroprotection. This is an important issue because it is well-established that inflammation is a crucial underlying mechanism of glial (macro- and micro-) activation and neuronal death in the diabetic retina. 

On this basis, the aims of the present study are: (1) to test the effect of topical administration of SST in a diabetes-induced model of retinal neurodegeneration, thus avoiding the potential confounding effects of STZ. For this purpose, we have used db/db mice, a spontaneous model of type 2 diabetes; (2) to further explore the anti-inflammatory mechanisms of SST in glial cells. This task was performed by using mouse retinal explants and cell cultures. 

## 2. Experimental Section

### 2.1. Animal Studies

Diabetic C57BLKS/J db/db male mice (BKS.Cg-Dock7m+/+LeprdbJ) and non-diabetic mice (db/+) were obtained from Charles River Laboratories, Inc. Blood glucose levels were measured from the blood obtained from the tail vein (glucose assay kit, Abbott, Chicago, IL, USA).

This study was approved by the Animal Care and Use Committee of VHIR (Vall d’Hebron Research Institute). All the experiments were performed in accordance with the tenets of the European Community (86/609/CEE) and ARVO (Association for Research in Vision and Ophthalmology).

#### 2.1.1. Topical ocular Treatment

SST-14 (2 mg/mL, 5 µL twice/daily, BCN peptides, Barcelona, Spain) (n = 10) or vehicle (phosphate buffered saline, 5 µL twice/daily) (n = 10) eye drops were administered directly onto the superior corneal surface of each eye using a micropipette in 8-week-old mice. Ten non-diabetic mice (db+) matched by age served as the control group. The treatment (SST-14 or vehicle) was administered twice daily for 15 days. At the end of the experimental protocol, mice were euthanized by cervical dislocation.

#### 2.1.2. Electroretinogram

Full-field electroretinography (ERG) recordings were measured using the Ganzfeld ERG platform (Phoenix Research Laboratories, Pleasanton, CA, USA) as reported elsewhere [14] and following ISCEV (International Society for Clinical Electrophysiology of Vision) recommendations [18].

#### 2.1.3. Immunohistochemical Analysis for Glial Activation Assessment

Macroglial (Müller cells) activation was evaluated by fluorescence microscopy using specific antibodies against GFAP (Glial fibrillar acidic protein) following the methodology described elsewhere [14]. The degree of glial activation was assessed using a scoring system based on the extent of GFAP staining [19] as previously reported [14,20].

Microglial activation was assessed by Iba-1 staining and analyzed by a semiquantitative score: (–) absence of positive cells for Iba-1/power field (20×); (+) scattered, 1–3 cell/power field (20×); (++) moderate, 4–10 cells/ power field (20×).

#### 2.1.4. Immunohistochemical Analysis for Apoptosis Assessment

TUNEL (Terminal Transferase dUTP Nick-End Labeling) coupled with fluorescein (DeadEnd Fluorometric TUNEL System kit, Promega, Madison, WI, USA) with Hoechst 33342, Trihydrochloride, Trihydrate (Molecular Probes) staining was used for apoptosis assessment. Sections of retina were permeabilized by incubation at room temperature for 5 min with 20 µg/mL Proteinase K solution freshly prepared. Green fluorescence (Alexa Fluor 594 goat-anti-rabbit (Invitrogen) (1:200 dilution prepared in PBS)) was used to identify apoptotic cells. For evaluation by fluorescence microscopy, an excitation wavelength in the range of 450–500 nm (i.e., 488 nm) and detection in the range of 515–565 nm (green) were used. The results are presented as percentage of TUNEL-positive cells with respect to the Hoechst staining cells obtained by Image J software.

#### 2.1.5. Glutamate Quantification

Glutamate was measured by reverse phase ultra-performance liquid chromatography (UPLC) (Acquity-UPLC, Waters, Milford, MA, USA) as aminoquinoline derivatives (AccQ-Tag chemistry, MassTrak AAA method and instruments, Waters, Milford, MA, USA), following a methodology previously described [21].

#### 2.1.6. Immunohistochemistry for GLAST

Glutamate-aspartate transporter (GLAST) was assessed by fluorescence microscopy using specific antibodies as previously reported [14].

### 2.2. In Vitro and Ex Vivo Studies

#### 2.2.1. Reagents and Antibodies

Fetal bovine serum (FBS) and culture media were obtained from Invitrogen (Grand Island, NY, USA). Bovine serum albumin (BSA) and crystal violet were purchased from Sigma-Aldrich (St Louis, MO, USA). Bacterial lipopolysaccharide (LPS) was purchased from Invivogen (Nucliber, Spain). Bradford reagent, acrylamide, immunoblot PVDF membranes and chemiluminescent HRP Substrate were purchased from Bio-Rad (Madrid, Spain). Antibodies against iNOS (sc-650) and phospho-p38α MAPK (Thr 180/Tyr182) (sc-17852-R) were purchased from Santa Cruz Biotechnology (Palo Alto, CA, USA). Anti-p38α MAPK (#9212) antibody was purchased from Cell Signaling Technology (Danvers, MA, USA). Anti-Arginase-1 (BD610708) antibody was purchased from BD Bioscience (Madrid, Spain). Anti-GFAP antibody (Z0334) was purchased from DAKO (Glostrup, Denmark) and anti-α-tubulin (T-5168) antibody was from Sigma-Aldrich (St Louis, MO, USA).

#### 2.2.2. Cell Culture

Mouse microglia Bv.2 cell line was provided by Dr. ML Nieto (IBGM, Spain). Bv.2 cells were cultured at 37 °C in a humidified atmosphere with 5% CO_2_ in RPMI supplemented with 10% (*v*/*v*) heat-inactivated FBS, 1% (*v*/*v*) penicillin/streptomycin (100 U/mL penicillin, 100 µg/mL streptomycin) and 2 mM L-glutamine (Gibco, Carlsbad, CA, USA). Cells were grown until 70% confluence was reached, washed twice with PBS and cultured in FBS-free RPMI medium for the indicated time periods. As pro-inflammatory stimuli we used: LPS (200 ng/mL), hypoxia (3% O_2_) or a diabetic milieu previously reported [22] (DM: 300 mM H_2_O_2_, 10 ng/mL IL-1β, 25 mM glucose) with or without a 4-h pre-treatment with SST (10-6 M).

#### 2.2.3. Analysis of the Cellular Viability by Crystal Violet Staining

After the treatments, the culture medium was removed and the attached cells were stained for 20 min with crystal violet (0.2% *w*/*v* in 2% ethanol). Plates were then rinsed with tap water, allowed to dry and followed by addition of 1% SDS to solubilize them. The absorbance of the samples was read spectrophotometrically at 560 nm.

#### 2.2.4. Analysis of Nitrites (NO_2_-)

Levels of NO_2_- were measured following the Griess method [23].

#### 2.2.5. Retinal Explants

Eight-week-old male C57BL/6J or db/db mice were sacrificed by cervical dislocation, eyes were enucleated and the lens, anterior segment, vitreous body, RPE and sclera were removed. For the experiments, the retinas were cultured in R16 medium (provided by PA Ekstrom, Lund University, Sweden). The retinal explants were pre-incubated with SST (10^−5^ M) for 4 h before the addition of stimuli. As pro-inflammatory stimuli we used conditioned medium (CM) collected from Bv.2 microglia cells pretreated or not with SST (10^−5^ M) and further stimulated with LPS (200 ng/mL) for 24 h. In other experiments, SST was directly added to Bv.2 cells or retinal explants for several time periods. All animal experimentation related to retinal explants was conducted in accordance with the regulations of the Association for Research in Vision and Ophthalmology (ARVO) and approved by the Animal Care and Use Committees of Spanish National Research Council (CSIC) and Comunidad de Madrid.

#### 2.2.6. Analysis of Reactive Gliosis Immunofluorescence in Retinal Explants

Retinal explants prepared from 8-week-old db/db mice and treated with SST (10^−5^ M) for 24 h were fixed in 4% paraformaldehyde (PFA) for 24 h at 4 °C and infiltrated with sucrose 25% (*w*/*v*). For immunofluorescence analysis, we followed the protocol previously described [24]. Retinas were incubated overnight in a humid chamber at 4 °C with rabbit anti-GFAP antibody in blocking solution. Samples were washed and incubated for 90 min with anti-rabbit antibody conjugated to Alexa 488 (1:2000; Molecular Probes, Eugene, OR, USA). After washing, sections were mounted with medium (Fluoromount G) containing 4-6-diamidino-2-phenylindole (DAPI). Staining was observed with an inverted laser confocal microscope LSM710 (Carl Zeiss Microscopy GmbH, Göttingen, Germany).

#### 2.2.7. Cytokine Detection Assay

TNF-α, IL-6, IL-1β, IL-4, IL-13 and IL-10 were determined in cell supernatants or serum using Luminex 100 IS technology (Merck Millipore, Darmstadt, Germany).

#### 2.2.8. Quantitative Real-Time PCR (RT-qPCR)

Total RNA was prepared with Trizol^®^ reagent (Invitrogen, Madrid, Spain) and reverse transcribed using a SuperScript™ III First-Strand Synthesis System (Invitrogen) following the manufacturer’s instructions. RT-qPCR was performed with an ABI 7900 sequence detector. Primer probe sets for mouse *Tnfa*, *Il6*, *Il1b*, *Il10*, *Nos2*, *Arg1* and *18s* were purchased as predesigned TaqMan gene expression assays (Applied Biosystems, Madrid, Spain).

#### 2.2.9. Western Blot

Retinal explants and Bv.2 microglial cells were homogenized in lysis buffer containing 50 mM Tris-HCl, 1% Triton X-100, 2 mM EGTA, 10 mM EDTA acid, 100 mM NaF, 1 mM Na_4_P_2_O_7_ and 2 mM Na_3_VO_4_ supplemented with protease inhibitors (10 μg/mL leupeptin, 10 μg/mL aprotinin and 100 μg/mL phenylmethylsulphonyl fluoride). Debris was removed by centrifugation at 14,000× *g* for 10 min at 4 °C and protein concentration was quantified using the Bio-Rad protein assay using BSA as a standard. Equivalent amounts of protein were resolved using denaturing sodium dodecyl sulphate-polyacrylamide gel electrophoresis (SDS-PAGE), followed by transfer to PVDF membranes. Blots were incubated for 2 h at room temperature or overnight at 4 °C with primary antibodies followed by 2 h incubation at room temperature with secondary antibodies. Finally, blots were developed with Clarity ECL Western Blot (BioRad, Madrid, Spain). Densitometry of the Western blots was performed using the ImageJ program.

### 2.3. Statistical Analysis

Data were analyzed by either one-way or two-way ANOVA followed by Bonferroni *t*-test or by unpaired *t*-test when comparisons were among two groups. Statistical tests were performed using SPSS 21.0 for Windows (SPSS Inc. IBM, Armonk, NY, USA). Differences were considered significant at *p* < 0.05.

## 3. Results

The evolution of blood glucose levels and body weight along the two weeks of treatment was similar in db/db mice treated with eye drops containing SST to the db/db mice treated with the vehicle.

### 3.1. Neurodegeneration Was Prevented in Retinas from db/db Mice Treated with SST Topically Administered

#### 3.1.1. Müller Glial Cells Activation

**Figure 1:**
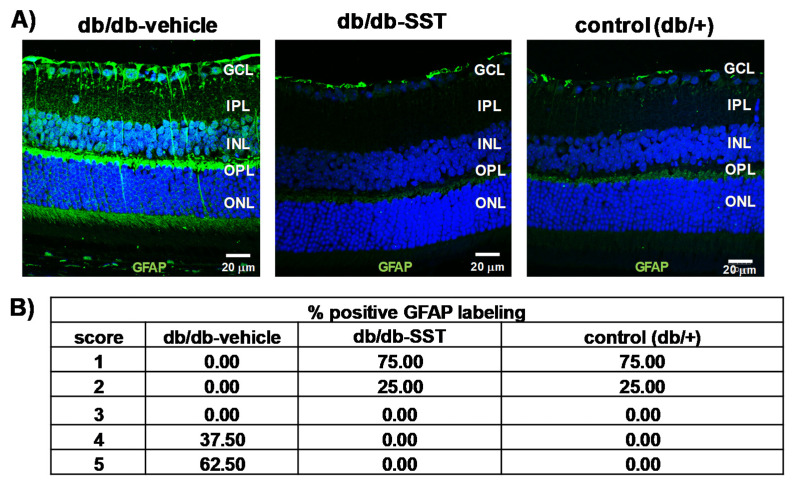
Effect of topical somatostatin (SST) administration on glial activation in db/db mice. (**A**) Comparison of GFAP immunoreactivity (green) in the retina among representative samples from a diabetic mouse treated with vehicle, a diabetic mouse treated with SST and a non-diabetic mouse. Nuclei were labeled with Hoechst (blue). ONL: outer nuclear layer; INL: inner nuclear layer; GCL: ganglion cell layer. Scale bars, 20 µm. (**B**) Quantification of glial activation based on the extent of GFAP staining. N: 10 mice per group.

The immunofluorescence of glial fibrillar acidic protein (GFAP) was restricted to the retinal ganglion cell layer (GCL) in non-diabetic mice (score 1–2). By contrast, diabetic mice treated with the vehicle presented a significantly higher extent of GFAP immunofluorescence than non-diabetic mice (Figure 1A,B). The administration of SST resulted in a significant decrease in glial activation, the GFAP score being <3 in all cases, similar to the observed in non-diabetic mice.

#### 3.1.2. Microglial Activation

**Figure 2:**
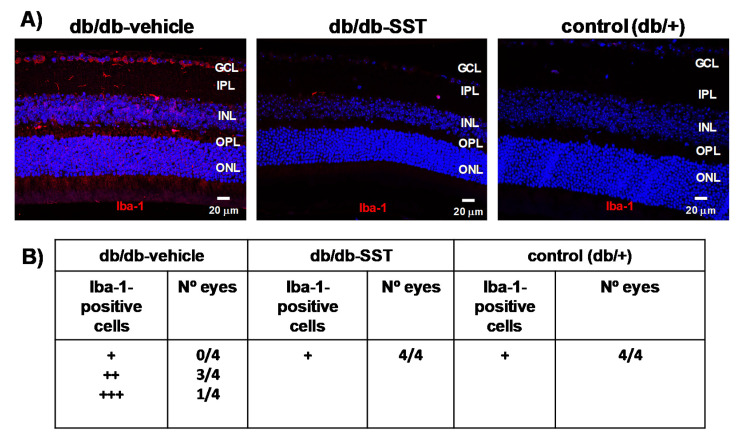
Effect of topical SST administration on microglia activation in db/db mice. (**A**) Representative immunofluorescence of Iba-1 (red) in representative mice from each group. Scale bars, 20 µm. (**B**) Semiquantitative assessment of microglial activation in the retina [-: absence of positive cells for Iba-1/power field (20×); +: scattered, 1–3 cell/power field (20×); ++: moderate, 4–10 cells/ power field (20×)]. N: 10 mice per group.

Iba-1-positive cells in the retina were higher in retinas from diabetic mice treated with the vehicle than in non-diabetic control mice (Figure 2A,B). In diabetic mice treated with SST, the number of Iba-1-positive cells was similar to that in non-diabetic mice.

#### 3.1.3. Apoptosis

**Figure 3:**
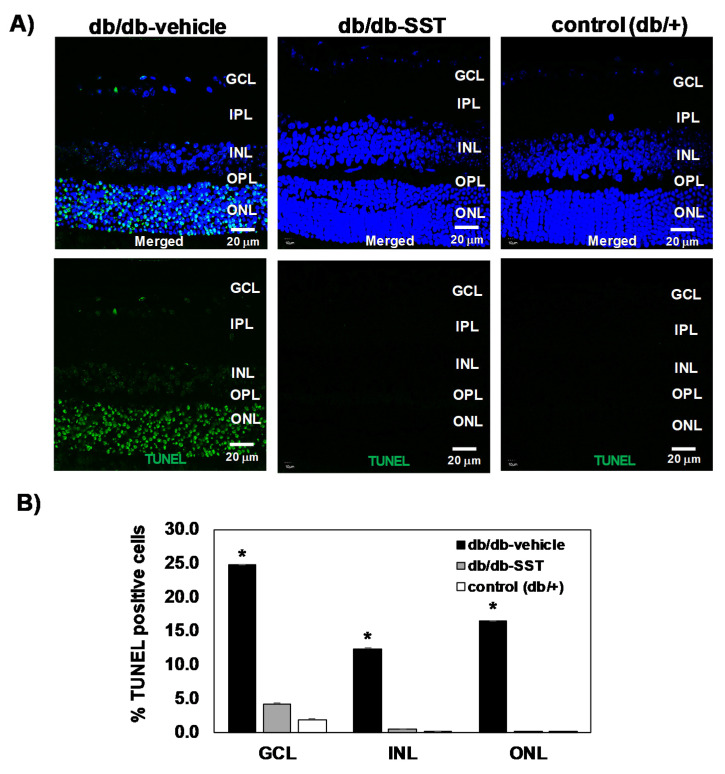
Effect of topical SST administration on apoptosis in retinas from db/db mice. (**A**) TUNEL-positive immunofluorescence (green) in a representative mouse from each group. Nuclei were labeled with Hoechst (blue). ONL: outer nuclear layer; INL: inner nuclear layer; GCL: ganglion cell layer. (**B**) Percentage of TUNEL-positive cells in the neuroretina. Black columns: db/db-vehicle; gray columns: db/db-SST; white columns: db/+. Results are mean ± SEM. * *p* < 0.01 in comparison with the other groups. N: 10 mice per group.

The percentage of apoptotic cells in retinas from db/db mice was significantly higher than in age-matched non-diabetic controls in all retinal layers (Figure 3). Diabetic mice treated with SST presented a significantly lower rate of apoptosis than diabetic mice treated with the vehicle. No differences in the percentages of apoptotic cells between diabetic mice treated with SST and non-diabetic mice were observed.

#### 3.1.4. ERG Abnormalities

**Figure 4:**
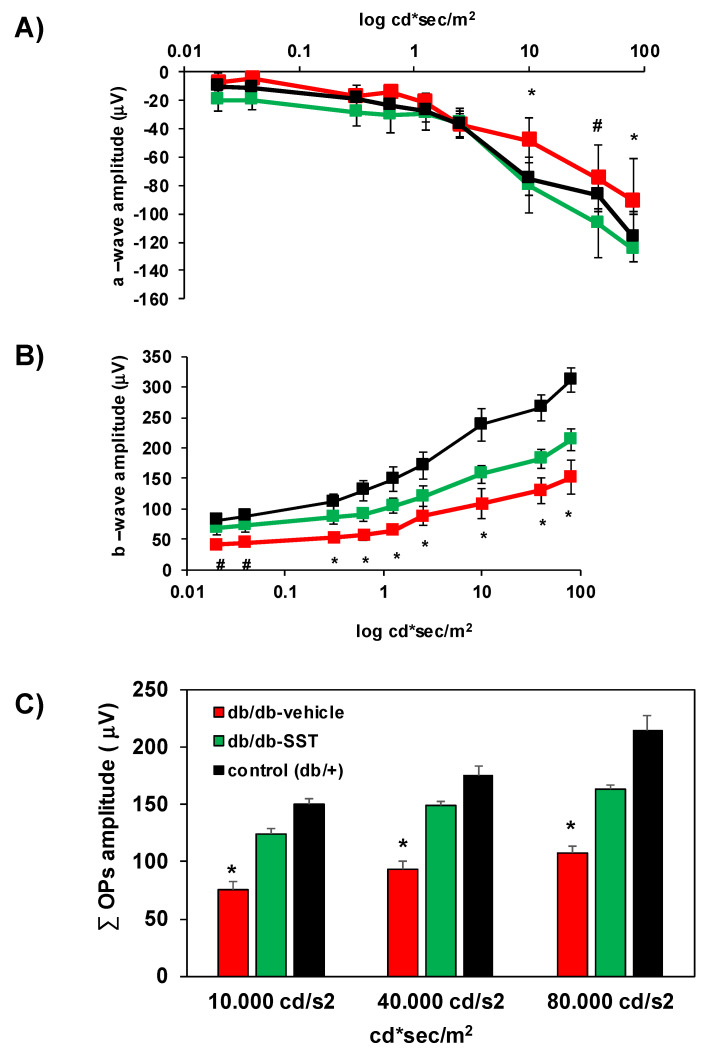
Effect of topical administration of SST on electroretinography (ERG) abnormalities in db/db mice. (**A**) Quantitative analysis of the amplitude of *a*-wave in db/db treated with vehicle (n = 10, red color), db/db treated with SST (n = 10, green color) and non-diabetic mice (n = 10, black color). (**B**) Amplitude of *b*-wave in db/db treated with vehicle (n = 10, red color), db/db treated with SST (n = 10, green color) and non-diabetic mice (n = 10, black color). (**C**) Oscillatory potentials (OPs) amplitude in the experimental groups. The first five OPs have been added up in performing this quantification. Results are mean ± SEM. * *p* < 0.05 (db/db treated with vehicle vs. the other groups). # *p* < 0.05 (db/db treated with vehicle vs. non-diabetic mice).

The *a*-wave (derived from the photoreceptor function) was significantly lower in diabetic mice treated with the vehicle than in non-diabetic mice (Figure 4A). The amplitude of the *b*-wave (predominantly produced by Müller and bipolar cells) was also significantly lower in diabetic mice in comparison with non-diabetic mice at all intensities (Figure 4B). Treatment with SST ameliorates these functional abnormalities induced by diabetes. In addition, SST treatment increases the amplitude of oscillatory potentials (OPs), which reflect the neuronal synaptic activity of amacrine cells and other neurons of the inner retina (Figure 4C).

### 3.2. SST Prevents the Increase of Glutamate Induced by Diabetes by Inhibiting GLAST Downregulation

**Figure 5:**
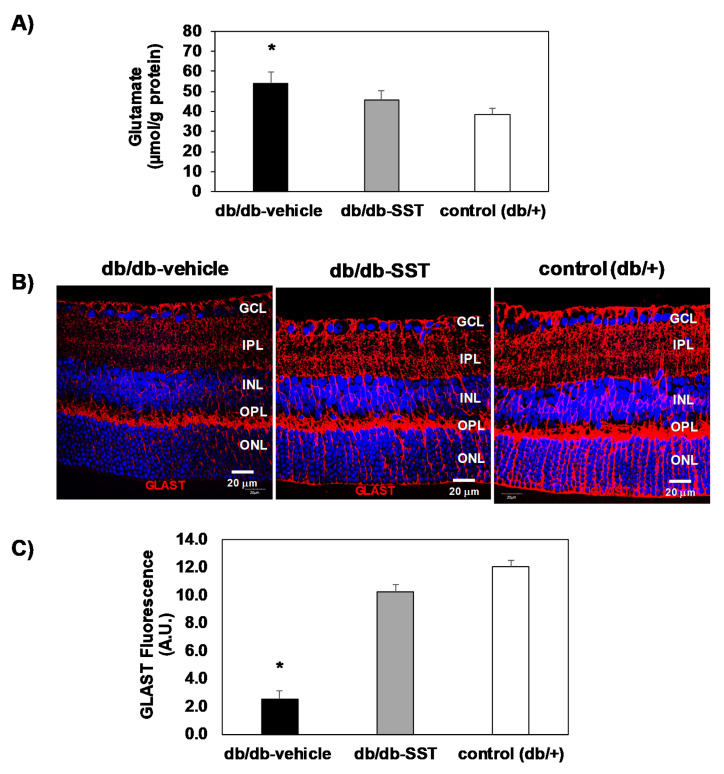
Effect of topical administration of SST in glutamate and GLAST levels in retinas from db/db mice. (**A**) Retinal concentration of glutamate measured by high-performance liquid chromatography in the experimental groups. * *p* < 0.01 in comparison with the other groups. (**B**) Comparison of GLAST immunofluorescence (red) among representative samples from each experimental group. Nuclei were labeled with Hoechst stain (blue). Scale bars, 20 µm. (**C**) Quantification of GLAST immunofluorescence in arbitrary units (A.U.). N = 10 mice per group. Results are the mean ± SEM. * *p* < 0.01 in comparison with the other groups.

Glutamate levels (μmol/g protein) in the diabetic retinas were higher than in non-diabetic retinas (Figure 5A). In diabetic mice treated with SST, glutamate concentrations were significantly lower in comparison with diabetic mice treated with the vehicle and similar to control mice. Furthermore, we observed that GLAST, the main glutamate transporter expressed by Müller cells, was significantly decreased in the retinas of diabetic mice treated with the vehicle in comparison with non-diabetic mice (Figure 5B,C). SST treatment significantly prevented the downregulation of GLAST induced by diabetes.

### 3.3. SST Prevented the Pro-Inflammatory Response in Bv.2 Cells Stimulated with LPS

**Figure 6:**
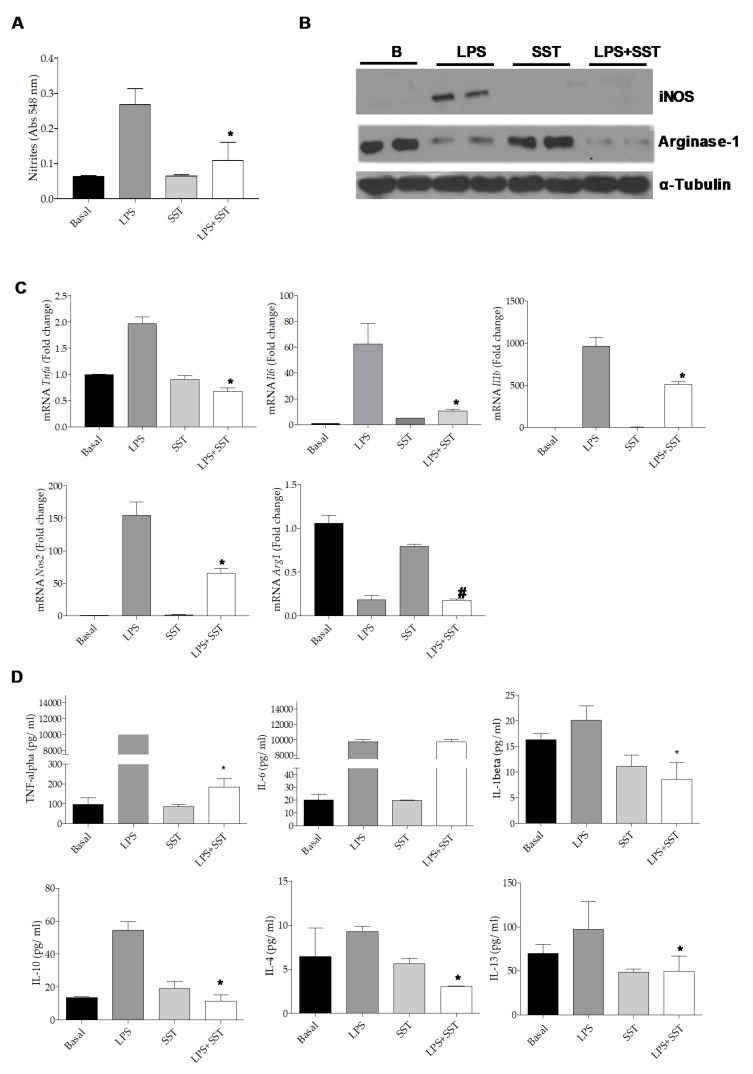
SST decreased inflammation in LPS-stimulated Bv.2 cells. Bv.2 microglial cells were pretreated with SST (10^−6^ M) for 4 h and then stimulated or not with LPS (200 ng/mL) for a further 24 h. (**A**) Levels of nitrites in the culture medium were analyzed and referred to the basal (untreated condition) levels. Colorimetric quantification was performed and results are mean ± SEM. The fold of change relative to the basal condition is shown (n = 6 independent experiments). * *p* ≤ 0.05 vs. LPS treatment (two-way ANOVA followed by Bonferroni *t*-test. (**B**) Protein extracts were analyzed by Western blot with antibodies against iNOS, Arginase-1 and α-Tubulin as loading control. Representative autoradiograms are shown. (**C**) *Tnfa*, *Il1b*, *Il6*, *Nos2* and *Arg1* mRNA levels were determined by RT-PCR. The fold of change relative to the basal condition is shown (n = 3 independent experiments). * *p* ≤ 0.05 vs. LPS treatment; # *p* ≤ 0.05 vs. SST (two-way ANOVA followed by Bonferroni *t*-test). (**D**) TNF-α, IL-1β, IL-6, IL-10, IL-4 and IL-13 released to the culture medium. Results are means ± SEM (n = 3 independent experiments). * *p* ≤ 0.05 vs. LPS treatment (two-away ANOVA followed by Bonferroni *t*-test).

We tested the effect of SST in LPS-mediated inflammation in Bv.2 microglial cells. Figure 6A shows that the pre-treatment for 4 h with 10^−6^ M SST, the concentration at which cellular viability was preserved (results not shown), decreased the release of nitrites (NO_2_-) to the culture medium induced by LPS. Likewise, iNOS, which was elevated in LPS-treated Bv.2 cells, decreased significantly in the presence of SST without changes in the LPS-mediated decrease in Arginase-1 protein content (Figure 6B and Supplementary Figure S1). Bv.2 cells treated with LPS plus SST also showed decreased expression of *Nos2*, *Tnfa*, *Il6* and *Il1b* mRNA levels (Figure 6C) and a reduced release of the pro-inflammatory cytokines TNF-α and IL-1β to the culture medium (Figure 6D). By contrast, levels of IL-6 in the culture medium were not decreased. Interestingly, LPS induced the release of the anti-inflammatory cytokine IL10 as previously reported [25], and this effect was attenuated in Bv.2 cells pre-treated with SST, suggesting less of a counteracting anti-inflammatory response. Of note, IL-4 and IL-13 levels were lower in LPS-stimulated Bv.2 cells pretreated with SST compared to the LPS condition.

The anti-inflammatory properties of the compound DSO2-ONJ, a member of the sp2-iminosugar glycolipid (sp2-IGL) family, have recently been reported [26]. This effect is mediated by direct p38α MAPK activation in microglial cells. In agreement with these data, treatment of Bv.2 cells with SST induced a rapid and marked p38α MAPK phosphorylation in a time-dependent manner (Supplementary Figure S2).

### 3.4. Decreased iNOS Expression in Retinal Explants Exposed to Conditioned Medium (CM) from LPS-Stimulated Bv.2 Microglial Cells Pre-Treated with SST

**Figure 7:**
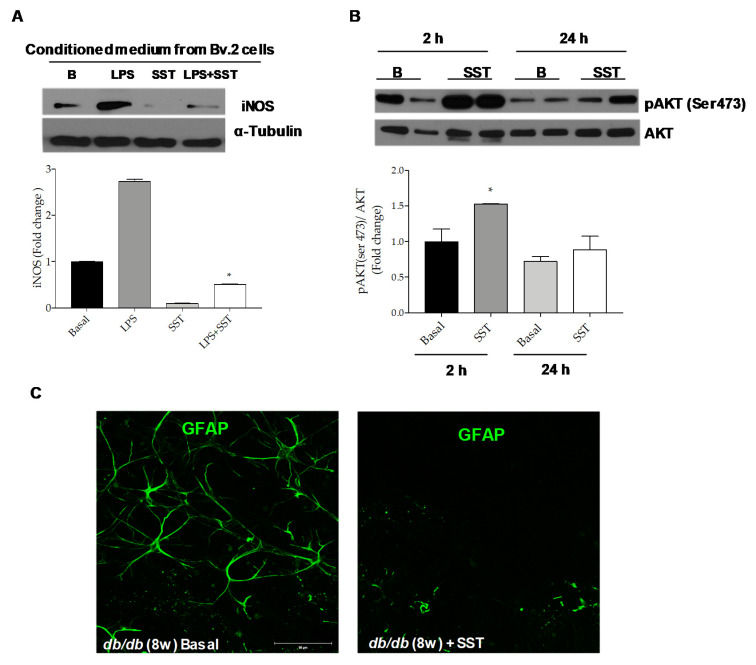
Beneficial effects of SST against inflammation in retinal explants. (**A**) Retinal explants from 8-week-old C57BL/6J mice were treated for 24 h with conditioned medium (CM) from LPS-stimulated Bv.2 cells in the absence or presence of SST (10^−5^ M) pre-treatment (4 h). Western blot was conducted with antibodies against iNOS and α-Tubulin as loading control. Representative autoradiograms are shown (n = 4 retinas per condition). (**B**) Retinal explants from 8-week-old C57BL/6J mice were treated for 2 or 24 h with SST (10^−5^ M) and analyzed by Western blot with antibodies against phosphorylated AKT (Ser473) and total AKT. Representative autoradiograms are shown. Blots were quantified by scanning densitometry and the results are mean ± SEM (n = 4 independent experiments). The fold change relative to the basal condition is shown. * *p* ≤ 0.05 vs. basal (two-way ANOVA followed by Bonferroni *t*-test). (**C**) Retinal explants from db/db mice at 8 weeks of age were treated with SST (10^−5^ M) (right panel) or vehicle (DMSO) (left panel) for 24 h. Immunostaining for GFAP (green) was carried out in whole retinas. Representative images are shown (n = 4 retinas per condition).

To get more insights on the benefit of the SST treatment under pro-inflammatory conditions in the retina, mouse retinal explants were cultured with conditioned medium (CM) from LPS-treated Bv.2 cells in the presence or absence of SST. As shown in Figure 7A, iNOS protein expression was increased in retinal explants exposed for 24 h to CM from LPS-treated Bv.2 cells and this effect was attenuated by the presence of SST.

Besides the effect of SST by counteracting the inflammatory potency of the CM in LPS-treated microglial cells, we also investigated the direct effect of SST in retinal explants. Figure 7B shows that addition of SST induced a rapid and marked increase in AKT Ser473 phosphorylation, a key mediator of pro-survival signaling in the human retina [27], as early as 2 h, suggesting a direct activation of pro-survival pathways. This effect declined at 24 h.

### 3.5. SST Decreases Reactive Gliosis in Retinal Explants from db/db Mice

Reactive gliosis detected in the retinas from diabetic db/db mice is maintained ex vivo in retinal explants [24], thereby offering a therapeutic opportunity in a preclinical setting. To further assess the anti-inflammatory effect of SST in DR, retinal explants from db/db mice were exposed to SST for 24 h followed by immunofluorescence analysis with the anti-GFAP antibody. As shown in Figure 7C and in agreement with the in vivo data (Figure 1), reactive gliosis was abolished in retinal explants from db/db mice treated with SST for 24 h.

### 3.6. SST Decreased Inflammatory Markers Induced by Hypoxia and a Defined Diabetic Milieu in Bv.2 Microglia Cells

**Figure 8:**
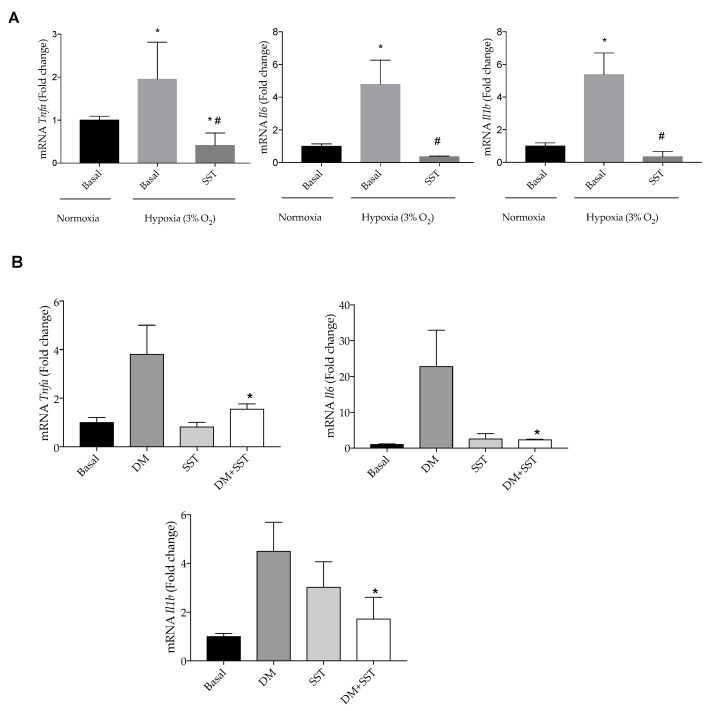
SST decreases pro-inflammatory markers induced by hypoxia and diabetic milieu in Bv.2 cells. (**A**) Effect of SST in the expression of pro-inflammatory parameters in hypoxia (3% O_2_)-stimulated Bv.2 microglial cells. Bv.2 cells were pre-treated for 4 h with SST (10^−6^ M) and then cultured under hypoxia (3% O_2_) for 24 h. mRNA levels of *Tnfa*, *Il1b* and *Il6* were determined by RT-PCR. The results are means ± S.E.M (n = 5 independent experiments). * *p* ≤ 0.05 vs. basal normoxia condition; # *p* ≤ 0.05 vs. basal hypoxia condition (one-way ANOVA followed by Bonferroni *t*-test. (**B**) Effect of SST in the expression of pro-inflammatory markers in Bv.2 cells stimulated with 25 mM glucose + 300 µM H_2_O_2_ + 20 ng/mL IL-1β (referred as diabetic milieu: DM). Bv.2 microglial cells were pre-treated for 4 h with SST (10^−6^ M) and then cultured under the diabetic environment of the DM for a further 24 h. *Tnfa*, *Il1b* and *Il6* mRNA levels were determined by RT-PCR. The fold change relative to the basal condition is shown (n = 3 independent experiments). * *p* ≤ 0.05 vs. diabetic milieu treatment (two-way ANOVA followed by Bonferroni *t*-test).

Finally, we tested the protective effect of SST against other pro-inflammatory insults that impact the retina during DR. Figure 8A,B show that SST decreased mRNA levels of pro-inflammatory cytokines induced by hypoxia (3% O_2_) or a combination of several pro-diabetic stimuli referred to as diabetic milieu (DM: 25 mM glucose + 300 μM H_2_O_2_ + 20 ng/mL IL-1β).

## 4. Discussion

DR was classically described as a microvascular disease. However, intensive research in the field has identified inflammation and neurodegeneration as early key events of DR pathogenesis. In the present study, we confirmed the neuroprotective effect of SST in the retina in db/db mice, a spontaneous mouse model of type 2 diabetes, thus avoiding the potential confounding effect of retinal toxicity in the experimental model of STZ-induced diabetes [11,12,13]. Furthermore, by using in vivo and in vitro approaches, we provide evidence that the neuroprotective effect of SST can be largely attributed to anti-inflammatory mechanisms.

Inflammation is a critical contributor to the development of DR [28,29,30]. In fact, we have previously reported that many inflammatory cytokines are increased in the vitreous fluid from diabetic patients with DR [31,32]. These inflammatory mediators pay an essential role in early neuronal cell death that occurs in the diabetic retina [30].

Müller cells, the principal glial cells of the retina which span its entire thickness, are known to be particularly vulnerable to damage. In diabetes, the Müller cells assume a reactive phenotype characterized by the upregulation of GFAP [33,34]. Since Müller glial cells are a significant source of numerous factors including inflammatory modulators, they play a significant role in the inflammatory process responsible for retinal damage induced by diabetes. In the present study, we found that the topical administration of SST in db/db mice or its addition to the culture medium of retinal explants prevented glial activation induced by diabetes and the downregulation of GLAST, thus reducing the extracellular concentration of glutamate and, consequently, ameliorating excitotoxicity and retinal neuronal death. In this regard, a significant reduction in neural apoptosis was observed in retinas from db/db mice treated with SST. Notably, these neuroprotective effects occurred in parallel with the improved function of the retina assessed by ERG.

Microglia are eye-specific cell types located in the inner and outer plexiform layers that become activated in response to stressors and migrate into the neuronal or photoreceptor layers where they may contribute to the inflammatory state [24,35,36]. The activation of microglia is determined by extracellular signals, including neuronal damage, chronic neurodegeneration, dying cells, extracellular liposaccharides and nucleic acids, which are recognized by a broad range of receptors, such as toll-like receptors (TLR) and receptors of advanced glycation end products [37]. Activated microglial cells not only act as scavengers, but also serve as rapid sensors of neuronal damage and are responsible for tissue repair and neural regeneration [38]. However, sustained neuroinflammation creates a proinflammatory milieu that may lead to the breakdown of the BRB and neural death [39]. In the present study, we found that SST prevented microglial activation in db/db mice, reinforcing its beneficial effect in targeting inflammation. We also performed molecular studies in microglial cells in order to assess the effect of SST in the inflammatory responses triggered by the immune cells of the retina. In this regard, we found that SST prevented the activation of the classical M1 response of Bv.2 microglial cells upon LPS stimulation as a potent pro-inflammatory trigger. This was evidenced by decreases in iNOS, nitrites and expression/secretion of pro-inflammatory cytokines. However, the anti-inflammatory effect of SST was not related to an enhanced M2 response since Arginase-1, a gold-standard M2 marker, remained at low levels in LPS-stimulated Bv.2 cells regardless of SST pretreatment. In addition, we did not detect elevations in the secretion of the anti-inflammatory cytokines IL-4/IL-13 that are associated with M2 microglia [24]. Notably, the anti-inflammatory effect of SST in Bv.2 cells was not only limited to LPS, but it was also observed in response to hypoxia. In this regard, it should be noted that hypoxia increases the transcription of genes encoding pro-inflammatory cytokines such as IL-6 via HIF-1α [40], as well as in Bv.2 cells exposed to a diabetic milieu previously reported to disrupt the integrity of the retinal pigment epithelium [41,42].

Of relevance is the direct activation of p38α MAPK by SST in Bv.2 microglial cells in a similar way reported by others with phosphatidylinositol ether lipid analogs (PIAs) and the alkyl phospholipid perifosine that displayed anti-inflammatory properties [43,44]. In the same line, our recent study with DSO2-ONJ, a member of the sp2-iminosugar glycolipid (sp2-IGL) family, showed that it was able to decrease inflammation in microglial cells and retinal explants through molecular mechanisms associated to the activation of p38α MAPK [26]. Therefore, further studies are necessary to decipher similarities in the mechanism of action between SST and these lipid-related compounds.

In an attempt to extend our findings in microglial cells to the context of inflammation in the whole retina, our results showed a marked reduction by SST in the inflammatory response of retinal explants exposed to the CM produced by LPS-stimulated Bv.2 cells. In this regard, we previously reported a protection by SST in human retinal pericytes against inflammation mediated by microglia [45]. Therefore, SST is able not only to mitigate neuroinflammation, but also to prevent the impairment of the neurovascular unit of the retina in a multifaceted manner. It is noteworthy to mention that in the present study we cannot discern that the decreased iNOS levels in the retinal explants are solely due to the action of the pro-inflammatory cytokines of the CM since a direct effect of LPS, which is also present, is likely affecting this response. In the case of CM collected from Bv.2 cells pretreated with SST before receiving LPS (LPS + SST), it is possible that, in addition to the lower pro-inflammatory potency of this CM, SST can also elicit a direct protective effect in the retinal explants.

As we previously reported, SST induced a potent protective effect against proapoptotic cascades in non-immune retinal cells such as photoreceptors cultured under high glucose through the activation of pro-survival signaling pathways [17]. In particular, the ability of SST to phosphorylate AKT in 661W photoreceptors and counteract anti-apoptotic pathways is likely a protective mechanism that prevents subsequent activation of the phagocytic M1 responses of microglial cells that trigger inflammation. In this regard, we have detected a rapid AKT phosphorylation in retinal explants from C57/BL6 mice treated with SST for 2 h that was maintained, although at lower levels, at 24 h. Consequently, in addition to its anti-inflammatory effect in the immune cells of the retina, SST directly triggers survival pathways that, as mentioned above, counteract proapoptotic pathways and protect from the collateral pro-inflammatory responses.

In terms of clinical translation, it should be noted that these results cannot be completely extrapolated to human beings. Indeed, in our experience, the db/db mouse model is very sensitive to both neuroprotective and anti-inflammatory agents [20,46,47,48], which could not be the case in humans. Another point to be considered is whether the dose of SST used in our in vitro experiments reflects what occurs in physiological conditions. In order to address this issue, we have calculated the dose of SST for treating cell cultures/explants taking into account the SST concentrations observed within the human vitreous fluid [5]. Nevertheless, it is very difficult to reproduce the retinal homeostasis in in vitro studies.

In conclusion, we confirmed that SST topically administered was able to prevent retinal neurodegeneration in db/db mice. In addition, SST directly triggers survival pathways in retinal explants. Furthermore, we found that SST prevented the activation of the classical M1 response of Bv.2 microglial cells upon LPS stimulation as a potent pro-inflammatory trigger. The anti-inflammatory effect of SST in Bv.2 cells was also observed in response to hypoxia. Overall, we provide evidence that the neuroprotective effect of SST can be largely attributed to anti-inflammatory mechanisms.

## Supplementary Materials

The following are available online at https://www.mdpi.com/2077-0383/9/8/2579/s1, Figure S1: Blots of Figure 6B were quantified by scanning densitometry and the results are mean ± S.E.M (n = 5 independent experiments). Figure S2: Direct effect of SST in the activation of p38α MAPK in microglial cells
