# Supplementary material for: Effect of Topical Administration of Somatostatin on Retinal Inflammation and Neurodegeneration in an Experimental Model of Diabetes
Source: J Clin Med. 2020 Aug 10;9(8):2579. doi: 10.3390/jcm9082579 (PMC7463891; doi:10.3390/jcm9082579)
Supplement: Supplementary file 1 [file jcm-09-02579-s001.pdf]

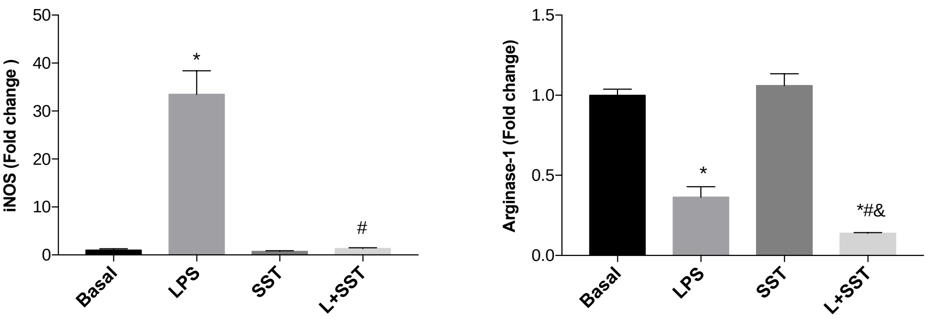

Figure S1. Blots of Figure 6B were quantified by scanning densitometry and the results are mean ± SEM (n = 5 independent experiments). The fold change relative to the Basal condition is shown. * $p \leq 0.05$ vs. Basal; # $p \leq 0.05$ vs. LPS; $ $p \leq 0.05$ vs. SST (two-way ANOVA followed by Bonferroni *t*-test).

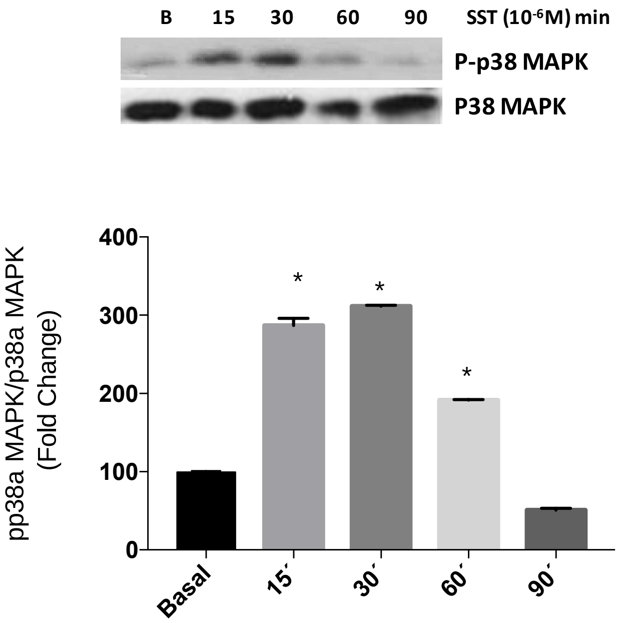

Figure S2. Direct effect of SST in the activation of p38α MAPK in microglial cells. Bv.2 cells were stimulated with 10^{-6} M SST for the indicated time-periods. Protein extracts were analyzed by Western blot with antibodies against phosphorylated p38α MAPK and p38α MAPK. Representative autoradiograms are shown. Blots were quantified by scanning densitometry, and the results are mean ± SEM (n = 3 independent experiments). The fold change relative to the Basal condition is shown. * $p \leq 0.05$ vs. Basal (untreated cells) (two-way ANOVA followed by Bonferroni *t*-test).
